# Erratum for Zhang et al., “Clinical and Molecular Characterizations of Carbapenem-Resistant Klebsiella pneumoniae Causing Bloodstream Infection in a Chinese Hospital”

**DOI:** 10.1128/spectrum.04453-22

**Published:** 2022-11-21

**Authors:** Na Zhang, Lihua Qi, Xiong Liu, Meiling Jin, Yuan Jin, Xiaojing Yang, Jiali Chen, Shiyu Qin, Fangni Liu, Yue Tang, Ruizhong Jia, Xiushan Zhang, Yong Wang, Jinpeng Guo, Jie Liu, Changjun Wang, Yong Chen

## ERRATUM

Volume 10, no. 5, e01690-22, 2022, https://doi.org/10.1128/Spectrum.01690-22. Page 1, abstract, line 14: “for ST11 CRKP” should read “for ST11 K. pneumoniae.”

Page 2, lines 12 to 8 from bottom: “The median age of 30 CRKP-BSI patients died during hospitalization, and 116 CRKP-BSI patients who survived during hospitalization were 61.5 years old (Inter Quartile Range [IQR]: 78 years old) and 47.5 years old (IQR: 53 years old). There were statistically significant differences in intensive care unit (ICU) admission, hospital stay, and use of urinary catheters between the 2 groups (*P < *0.05)” should read “The median hospital stays of 30 CRKP-BSI patients who died during hospitalization and 116 CRKP-BSI patients who survived during hospitalization were 61.5 days (inter quartile range [IQR]: 78 days) and 47.5 days (IQR: 53 days). There were statistically significant differences in intensive care unit (ICU) admission and use of urinary catheters between the 2 groups (*P < *0.05).”

Page 3, Table 2, column 4, row 2: “0.036” should read “0.037.”

Page 9, line 15 from bottom: “totaled 5.6 Mb” should read “averaged 5.6 Mb.”

